# Peptide G-Protein-Coupled Receptors and ErbB Receptor Tyrosine Kinases in Cancer

**DOI:** 10.3390/biology12070957

**Published:** 2023-07-04

**Authors:** Terry W. Moody, Irene Ramos-Alvarez, Robert T. Jensen

**Affiliations:** Center for Cancer Training, NCI, and Digestive Diseases Branch, NIDDK, NIH, Bethesda, MD 20892, USA; irene.ramosalvarez@nih.gov (I.R.-A.); robert.jensen@nih.gov (R.T.J.)

**Keywords:** G-protein-coupled receptor, receptor tyrosine kinases, transactivation, tyrosine kinase inhibitors, monoclonal antibodies, cancer

## Abstract

**Simple Summary:**

Cancer growth is regulated by receptor tyrosine kinases (RTKs) and G-protein-coupled receptors (GPCRs). The epidermal growth factor receptor (EGFR) is an RTK that binds ligands and causes tyrosine phosphorylation of protein substrates. Alternatively, the EGFR can tyrosine itself, leading to the activation of the ERK pathway, increasing cellular proliferation. The EGFR is mutated in several lung cancer patients, and these patients can be treated with tyrosine kinase inhibitors. The neurotensin receptor (NTSR1) is a GPCR, which binds peptides and alters signal transduction. Adding neurotensin to lung cancer cells increases phosphatidylinositol turnover, resulting in elevating cytosolic Ca^2+^ and activating protein kinase C. SR48692 is an NTSR1 antagonist, which inhibits lung cancer proliferation. RTKs and GPCRs can act independently to alter cancer growth; however, NTSR1 regulates the tyrosine phosphorylation of RTKs such as the EGFR. RTKs and GPCRs interact to increase cancer proliferation.

**Abstract:**

The ErbB RTKs (EGFR, HER2, HER3, and HER4) have been well-studied in cancer. EGFR, HER2, and HER3 stimulate cancer proliferation, principally by activating the phosphatidylinositol-3-kinase and extracellular signal-regulated kinase (ERK) pathways, resulting in increased cancer cell survival and proliferation. Cancer cells have high densities of the EGFR, HER2, and HER3 causing phosphorylation of tyrosine amino acids on protein substrates and tyrosine amino acids near the C-terminal of the RTKs. After transforming growth factor (TGF) α binds to the EGFR, homodimers or EGFR heterodimers form. HER2 forms heterodimers with the EGFR, HER3, and HER4. The EGFR, HER2, and HER3 are overexpressed in lung cancer patient tumors, and monoclonal antibodies (mAbs), such as Herceptin against HER2, are used to treat breast cancer patients. Patients with EGFR mutations are treated with tyrosine kinase inhibitors, such as gefitinib or osimertinib. Peptide GPCRs, such as NTSR1, are present in many cancers, and neurotensin (NTS) stimulates the growth of cancer cells. Lung cancer proliferation is impaired by SR48692, an NTSR1 antagonist. SR48692 is synergistic with gefitinib at inhibiting lung cancer growth. Adding NTS to lung cancer cells increases the shedding of TGFα, which activates the EGFR, or neuregulin-1, which activates HER3. The transactivation process is impaired by SRC, matrix metalloprotease, and reactive oxygen species inhibitors. While the transactivation process is complicated, it is fast and occurs within minutes after adding NTS to cancer cells. This review emphasizes the use of tyrosine kinase inhibitors and SR48692 to impair transactivation and cancer growth.

## 1. Introduction

Receptor tyrosine kinases (RTKs) of the erbB family are large proteins (approximately 1200 amino acids) that are important in regulating cancer proliferation. These include the epidermal growth factor receptor (EGFR or ErbB1), HER2 (ErbB2), HER3 (ErbB3), and HER4 (ErbB4) [1]. When the EGFR is activated by ligands such as transforming growth factor (TGF) a, protein substrates, such as phospholipase C and phosphatidylinositol-3-kinase, are tyrosine phosphorylated, leading to increased cancer proliferation and survival [2,3]. The RTKs mediate the following reaction:

Protein-OH + MgATP → Protein-OPO3^−^ + MgADP + H^+^.

The EGFR tyrosine phosphorylation is impaired by tyrosine kinase inhibitors (TKIs), such as gefitinib or osimertinib [4], which impair ATP binding to the RTK. The EGFR is an oncogene in lung cancer that is frequently mutated increasing protein kinase activity [5]. Lung cancer patients with EGFR mutations can be treated with gefitinib or osimertinib. HER2 is an orphan receptor with no known ligands; however, it forms heterodimers with the EGFR stimulating its protein kinase domain [6]. HER2 is inhibited by the TKI lapatinib or the monoclonal antibody (mAb) trastuzumab (Herceptin) [7,8,9]. Breast cancer patients with HER2 overexpression are often treated with Herceptin. HER3 is activated by neuregulin-1 or -2; however, HER3 has weak protein kinase activity [10]. HER3 protein kinase activity is increased when it forms heterodimers with HER2 or other ErbB RTK members [11]. While the EGFR, HER2, and HER3 stimulate cancer proliferation, the role of HER4 is more complex. HER4 has four splice variants that can stimulate or inhibit cancer growth [12,13]. HER4 is stimulated by neuregulins-1, -2, -3, or -4 [1] but inhibited by TKIs, such as ibrutinib or mAbs [14,15]. RTKs are impaired by TKIs and mAbs, which reduce tyrosine phosphorylation.

G-protein-coupled receptors (GPCRs) are proteins of moderate size (approximately 400 amino acids), which are embedded in the plasma membrane [16]. While RTKs cross the plasma membrane once, the GPCRs have seven transmembrane domains. GPCRs have an extracellular NH_2_ terminal and an intracellular C-terminal. In addition, there are three extracellular loops that can interact with ligands and three intracellular loops that can interact with G proteins. The GPCRs alter signal transduction. Peptides such as vasoactive intestinal peptide increase cAMP after interacting with the G protein Gs, which is composed of Gas and Gbg subunits [17]. The Gas subunit stimulates adenylyl cyclase, causing the metabolism of ATP to cycle AMP, which activates protein kinase A, causing serine and threonine phosphorylation of protein substrates. The bg subunits activate phosphatidylinositol-3-kinase and SRC [18]. Lung cancer growth is increased by VIP and decreased by the antagonist VIPhybrid [19]. VPAC1 is a class B secretin-like GPCR, whereas the peptide neurotensin (NTS) binds to class A rhodopsin-like GPCR. NTSR1 interacts with Gaq, which stimulates phospholipase C [20], changing the metabolism of phosphatidylinositol-4,5-bisphosphate into inositol tris-phosphate, which elevates cytosolic Ca^2+^ [21]. Another phospholipase C product is diacylglycerol, which activates protein kinase C [22]. Adding NTS to lung cancer cells stimulates their proliferation, which is impaired by SR48692 [23]. GPCRs are inhibited by antagonists, which block ligand binding.

GPCRs regulate the tyrosine phosphorylation of the EGFR and other ErbB RTKs via transactivation [24]. Transactivation can occur through a ligand-dependent or ligand-independent process [25]. In the ligand-dependent pathway, NTS increased the metabolism of precursor proteins, resulting in the shedding of TGFa from the plasma membrane into the extracellular fluids where it binds to the EGFR. After dimerization, the phosphorylation of the EGFR at tyrosine^1068^ activates ERK, causing increased proliferation [26]. In the ligand-independent pathway, SRC is activated, causing EGFR phosphorylation at tyrosine^845^ [27]. Both pathways are very fast, and the EGFR is phosphorylated within 1 min after ligand addition. The tyrosine phosphorylation is transient, and within 1 h, phosphatase enzymes remove phosphate from the EGFR. Reactive oxygen species (ROS) are required for transactivation [28]. GPCRs stimulate NADPH oxidase, which produces ROS [29]. NTSR1 regulates the transactivation of the EGFR, HER2, and HER3, which is inhibited by GM60001 (matrix metalloprotease inhibitor), PP2 (SRC inhibitor), and N-acetylcysteine (antioxidant) [30,31,32]. Transactivation is an important way in which GPCRs and RTKs regulate cancer growth.

## 2. RTKs

**Figure 1:**
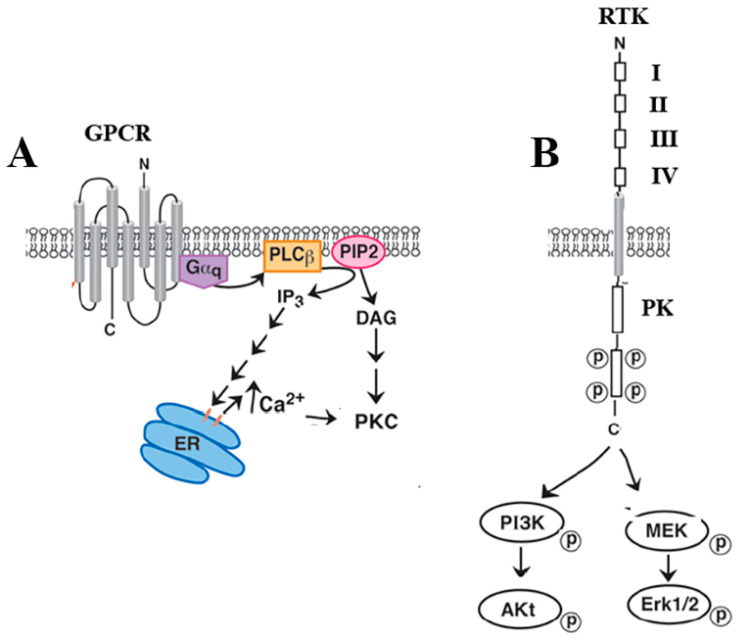
GPCR and RTK signal transduction. (**A**) GPCRs such as NTSR1 interact with Gq, increasing phospholipase (PL)C activity, resulting in the metabolism of phosphatidylinositol bisphosphosphate (PIP_2_). The resulting inositol-3-phosphate (IP_3_) releases calcium (Ca^2+^) from the endoplasmic reticulum (ER), whereas the diacylglycerol (DAG) increases protein kinase (PK)C activity. (**B**) Near the N-terminal, the EGFR has extracellular domains I and III, which participate in ligand binding, whereas domain II participates in dimerization. Increased protein kinase (PK) activity leads to phosphorylation of tyrosine amino acids near the RTK C-terminal, resulting in the phosphorylation of ERK and/or AKT.

Figure 1 shows that the ErbBRs have an extracellular N-terminal domain with a ligand binding site, followed by a single transmembrane domain and a C-terminal intracellular domain with protein kinase activity [1]. The N-terminal contains approximately 640 amino acids. The extracellular EGFR is divided into domains I and III, which bind growth factors and are enriched in leucine amino acids, whereas domains II and IV are enriched in cysteine amino acids. Domain II is important in forming ErbBR homodimers and heterodimers. The extracellular domain is followed by a transmembrane domain of approximately 23 amino acids and an intracellular domain of approximately 550 amino acids. The intracellular domain contains a juxtamembrane segment, protein kinase domain, and C-terminal that has tyrosine amino acids, which can be phosphorylated. In contrast, GPCRs such as NTSR1 alter signal transduction (Figure 1).

**Figure 2:**
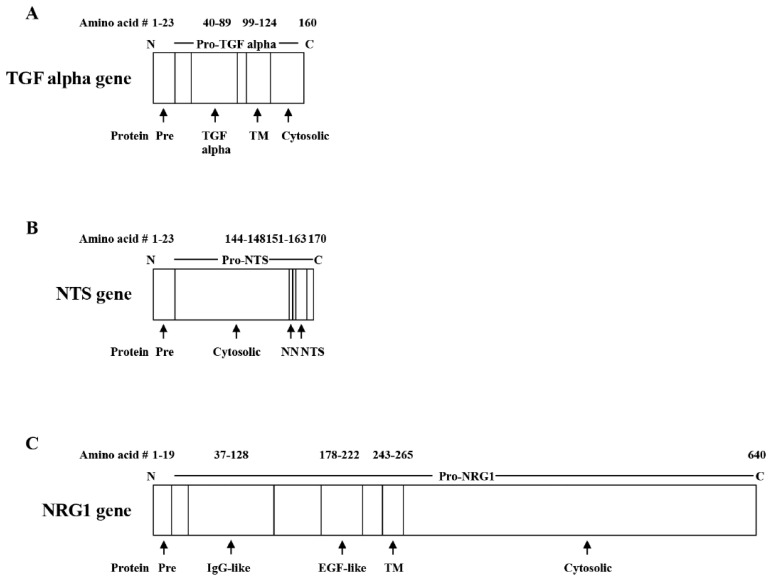
Growth factors. (**A**). TGF alpha is derived from a 160-amino-acid precursor protein. After removal of the pre- (N-terminal signal peptide), pro-TGFα contains TGFα, a transmembrane (TM) domain, and a cytosolic domain. (**B**) NTS is derived from a 170-amino-acid precursor protein. After removal of the N-terminal signal peptide, pro-NTS contains a cytosolic domain, neuromedin N (NN), and NTS. (**C**) Neuregulin-1 (NRG1) is derived from a 640-amino-acid precursor protein. After removal of the signal peptide, pro–NRG1 contains an immunoglobulin (IgG)-like domain, an EGF-like domain, a TM domain, and a cytosolic domain.

Growth factors bind to the EGFR, HER3, and HER4 [33]. Growth factors for the EGFR include amphiregulin, betacellulin, epidermal growth factor, epigen, epiregulin, heparin binding epidermal growth factor, and TGFα. The growth factors are synthesized as precursor proteins that have a single transmembrane domain. Figure 2 shows that TGFα is synthesized as a 160-amino-acid precursor protein. Removal of the N-terminal 23 amino acids through a signal protease generates pro-TGFα, which contains a transmembrane domain and is metabolized by enzymes to a soluble 50-amino-acid form, which binds the EGFR with high affinity [34]. The EGFR ligands are enriched in cysteine amino acids and contain three disulfide bonds. Ligands for HER3 include neregulin-1 and -2. Figure 2 shows that the N-terminal 19 amino acids of preproneuregulin-1 are removed by a signal protease. Proneuregulin-1 is metabolized by β-secretase to a soluble 222-amino-acid form [35]. Neuregulin-1 contains an IgG-like domain and EGF-like domain, which binds with high affinity to HER3 and HER4. Neuregulin-1 can undergo alternative splicing to 6 different proteins and 31 isoforms. In lung cancer patients, 1% have neuregulin-1 fused to CD44 [36]. Growth factors for HER4 include neuregulin-1, -2, -3, and -4, epiregulin, HB-EGF, and betacellulin. The following sub-sections discuss the activation of the EGFR, HER2, HER3, and HER4.

### 2.1. EGFR

The EGFR is important in the development of normal mammals. Null mutations of the EGFR in mice result in embryonic lethality [37]. Knockout of the EGFR gene in mice results in lung, gastrointestinal, and skin defects. The EGFR is overexpressed in many cancers relative to normal tissues, including breast cancer, colorectal cancer, esophageal cancer, head and neck cancers, glioblastoma, and lung cancer [38,39,40]. Lung cancer kills approximately 154,000 citizens in the U.S.A. and 2.1 million in the world annually [41]. It is associated with cigarette smoking and is treated traditionally with chemotherapy; however, the 5-year survival rate is only 16% [41]. Most lung cancers are detected after they have undergone metastasis from the lung to the brain, bone, pancreas, and/or liver. Lung cancer comprises SCLC, a neuroendocrine tumor, and NSCLC, an epithelial tumor. NSCLC is present in 80% of lung cancer patients, and adenocarcinoma of the lung is most common. Recently, NSCLC patients benefited from treatment with immune checkpoint inhibitors [42,43]. NSCLC cells have EGFR mutations in the protein kinase domain, and the mutations occur in approximately 10% of Caucasian and 35% of Asian NSCLC patients [44]. If the tumor has L^858^R EGFR mutations or exon 19 deletions, lung cancer patients can be treated with TKIs, erlotinib, or gefitinib. Unfortunately, after a year, the patients become resistant to erlotinib or gefitinib due to further lung cancer mutations, such as T^790^M EGFR [45]. Osimertinib is an irreversible EGFR TKI that is used to treat patients with T^790^M mutations. Osimertinib interacts with cysteine^797^, impairing ATP binding [46]. Unfortunately, resistance to osimertinib occurs with further C^797^S EGFR mutations. There is a need to develop additional TKIs for NSCLC patients with new EGFR mutations. Glioblastoma has deletions in the EGFR extracellular domain (EGFRvIII) [47]; however, patients with GBM respond to temozolomide (chemotherapeutic) but not TKIs.

The EGFR contains 1210 amino acids, and extracellular domains I and III bind TGFα with high affinity (Figure 1). When ATP is metabolized to ADP, an amino acid with a hydroxyl group, e.g., tyrosine, is phosphorylated [4]. After growth factor binding, the EGFR changes from a tethered to extended conformation. Extracellular domain II facilitates the forming of EGFR homodimers or heterodimers [1]. The extended EGFR conformation has elevated protein kinase activity, increasing to the phosphorylation of phospholipase C and STAT. Phospholipase C alters phosphatidylinositol turnover, whereas STATs dimerize and alter gene transcription in the nucleus [48]. The EGFR can be phosphorylated at 10 different tyrosine amino acids such as tyrosine^1068^, leading to the activation of ERK. Phosphorylated ERK enters the nucleus and alters gene expression, leading to increased cellular proliferation. The lung cancer EGFR can be treated with TKIs. In addition, the mAbs cetuximab and necitumumab prevent ligand binding to the EGFR [4]. Cetuximab is used to treat certain types of colon cancer, whereas necitumumab is used to treat certain types of lung cancer.

### 2.2. HER2

HER2 is important in the development of the heart, and HER2 knockout mice develop dilated cardiomyopathy [49]. HER2 is overexpressed or amplified in breast cancer and ovarian cancer [50]. Breast cancer is diagnosed in 234,000 American women each year and 2.1 M in the world [1]. Because breast cancer is often detected early before tumor metastasis, there are numerous therapies for breast cancer, and the 5-year survival rate is approximately 90%. Treatments include surgical resection, trastuzumab (mAb) for patients with overexpressed HER2, docetaxel (chemotherapeutic), and lapatinib (TKI) [51]. If the breast cancer cells express estrogen receptors or progesterone receptors, the patients can be treated with tamoxifen. It is difficult to treat triple-negative breast cancer patients due to the lack of HER2, progesterone receptors, and estrogen receptors. HER2 is mutated in approximately 2% of breast cancer patients. Mutations can occur at codons 309 or 310 of the extracellular domain [52]. G^309^A-HER2 mutations increase tumor formation due to excessive formation EGFR/HER2 heterodimers.

HER2 (1255 amino acids) is larger than the EGFR, but it exists in an extended form. In contrast, the EGFR exists in a tethered conformation where it can bind growth factors. After binding, the EGFR changes to the extended form where domain II facilitates dimerization. In HER2, there is no room for growth factors to bind between domains I and III. To increase protein kinase activity, HER2 must form heterodimers with the EGFR, HER3, and/or HER4 [53]. These heterodimers form through extracellular domain II interactions. HER2 is phosphorylated at eight tyrosine amino acids including tyrosine^1248^, activating the ERK pathway, leading to increased cellular proliferation. Even though numerous therapies are available for breast cancer patients, approximately 41,000 patients die annually from this disease in the U.S.A. New therapies are needed for triple-negative breast cancer patients, and the role of immune checkpoint inhibitors is being investigated.

### 2.3. HER3

Knockout of the HER3 gene results in embryonic lethality [54] and impaired development of the ductal gland in the breast [55]. HER3 binds neuregulin-1 and -2; however, it has weak protein kinase activity [56]. When it forms heterodimers with HER2, 11 tyrosine amino acids near the C-terminal of HER3 can be phosphorylated, and tyrosine^1289^ causes phosphorylation of phosphatidylinositol-3-kinase [57]. HER3 (1342 amino acids) is overexpressed in many cancers, especially NSCLC [58]. HER3 expression is associated with increased tumor angiogenesis and metastasis. Patients whose tumors express HER3 have a 1.6-fold higher death risk than patients who are HER3-negative [59]. The G^284^R-HER3 mutation increases the growth of cancer cells that express HER2 [60]. Numerous mAbs have been made against HER3. Seribantumab blocks neuregulin-1 binding to HER3 and increases the survival of patients with high neuregulin-1 levels [61]. Patritumab inhibits HER3 growth factor binding and is being tested with erlotinib to treat patients with advanced NSCLC [62]. Numerous HER3 mAbs are being developed for cancer treatment.

HER3 expression confers resistance to many therapeutic agents [63]. NSCLC patients who become resistant to TKIs have increased HER2/HER3 dimerization [64]. In breast cancer, tumor resistance to trastuzumab is driven by HER3 bypass signaling [65]. Patients with HER2/HER3 heterodimers are resistant to camptothecin, etoposide, and paclitaxel [66]. Because cancers often have multiple ErbB family members, Pan-HER strategies have been developed for treatment. Pan-HER (Sym013) is a mixture of six antibodies targeting EGFR, HER2, and HER3 [67]. Unfortunately, a clinical trial failed using Pan-HER due to excessive drug toxicity. Other studies are in progress using combination inhibitors for the EGFR, HER2, and HER3 [68].

### 2.4. HER4

HER4 has both growth stimulatory and growth inhibitory properties, whereas the EGFR, HER3, and HER2 stimulate cancer growth [69]. HER4 expression is downregulated in some aggressive tumors [70]. HER4 is essential in normal development and HER4-null mice die from defective heart development [71]. HER4 has a ligand binding site and protein kinase activity. Neuregulin-1 -2, -3, and -4 are ligands for HER4. Abnormal neuregulin-1 signaling is associated with schizophrenia [72]. HER4 (1308 amino acids) has 18 tyrosine amino acids, which can be phosphorylated, and phospho-tyrosine^1284^ activates the ERK pathway. Ibrutinib binds irreversibly to cysteine^803^ of HER4, blocking ATP binding and reducing the growth of lung cancer cells enriched with WNT5A [14]. Other TKIs for HER4 include afatinib, dacomitinib, and neratinib [73]. An advantage of afatinib, dacomitinib, and neratinib is that they block the EGFR and HER2, in addition to HER4.

HER4 but not the EGFR, HER2, or HER3 undergoes alternative splicing. An extracellular splicing site generates JMa or JMb [74]. An intracellular alternative splicing site generates Cyt1 or Cyt2 [75]. When HER4 binds neuregulin-1, tumor necrosis-alpha converting enzyme (TACE) is activated, metabolizing JMa but not JMb [76]. Cyt2 lacks HER4^1046–1061^ and cannot activate phosphatidylinositol-3-kinase, whereas Cyt1 can increase cell survival [77]. JMa-Cyt1 and JMa-Cyt2 are metabolized by γ-secretase to soluble intracellular domains, which activates STAT5, altering gene expression. Depending on how HER4 is metabolized, it can inhibit the proliferation of certain breast cancer cells or stimulate growth [78]. HER4 function results from a signal input layer (growth factors), a signal transformation layer (protein kinase), and intracellular pathways, including ERK, AKT, and STAT.

## 3. GPCRs

**Figure 3:**
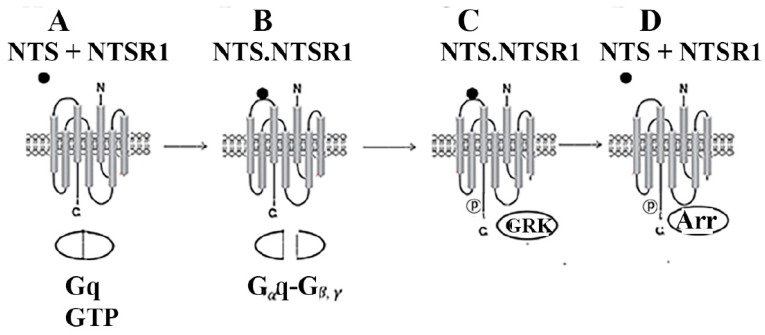
NTSR1 processing. (**A**) When NTS (●) binds to NTSR1 Gq binds GTP. (**B**) Subsequently, Gq dissociates to Gαq + Gβγ. (**C**) NTSR1 is serine phosphorylated by GRK. (**D**) β-Arrestin (Arr) recognizes the phosphorylated NTSR1 causing ligand dissociation.

GPCRs contain an extracellular N-terminal of approximately 100 amino acids, 7 transmembrane domains each of about 20 amino acids, and a C-terminal of approximately 100 amino acids, which are intracellular [79]. There are three extracellular (EC) loops, which can participate in ligand binding, and three intracellular (IC) loops, which can participate in G-protein interactions. When an agonist binds the GPCR, an outward movement of transmembrane V and VI occurs, creating a cavity of the cytosolic side in which the G protein docks [80]. Figure 3A shows that when NTS binds to NTSR1, Gq binds GTP. The GPCR undergoes a conformation change, and Gαq dissociates from Gβγ (Figure 3B). When NTS binds to NTSR1, Gαq is activated, causing phosphatidylinositol turnover within seconds. The NTSR1 causes transactivation of the EGFR within minutes. Figure 3C shows that GPCR signaling is desensitized after G-protein kinases phosphorylate NTSR1 [81]. Subsequently, P-NTSR1 forms a complex with β-Arrestin, causing signal termination [82] (Figure 3D). The GPCR is packaged into the endosome where it can be recycled to the plasma membrane or degraded in the lysosome [83].

The 13-amino-acid NTS is derived from a precursor protein (170 amino acids). Figure 2 shows that the signal peptide at the N-terminal is removed, resulting in a 147-amino-acid-long fragment NTS (LF-NTS) [84]. Pro-NTS or LF-NTS, which is present in cancer patient sera, is neutralized by mAb. LF-NTS activates NTSR1 and increases P-EGFR, P-HER2, and P-HER3. LF-NTS mAb reduces lung cancer proliferation and increased sensitivity to chemotherapy [85]. Prohormone convertases metabolize proNTS to neuromedin N (NN) or NTS, which binds to NTSR1. Upon release from brain neurons, NTS has significant interaction with the dopaminergic system and causes luteinizing hormone and prolactin release from the normal pituitary [86]. NTS binds with high affinity to NTSR1 and NTSR2, which interact with Gαq and cause PIP_2_ turnover [87]. NTSR1 contains 418 amino acids. The C-terminal of NTS (NTS^8–13^) is essential for high-affinity binding to NTSR1, and SR48692 is a small-molecule antagonist [88]. The structure of NTSR1 has been determined, and NTS^8–13^ localizes to the top of a binding pocket and interacts with transmembrane domains 6 and 7 as well as extracellular loops 2 and 3. SR48692 binds deep into the binding pocket and prevents NTS^8–13^ from binding with high affinity [88].

NTSR1 mRNA is present in lung cancer cells but not NTSR2 mRNA. Adding NTS to lung cancer cells increases phosphatidylinositol–bisphosphate metabolism as well as Rho GTPase, NFκB, and ERK activity. The phosphorylated ERK increases the expression of the nuclear oncogenes c-fos and c-jun, leading to increased proliferation [89]. Adding NTS to lung cancer cells increases WNT signaling, leading to epithelial to mesenchymal transitions [90]. NTS stimulates the growth of numerous cancers [91]. NTS and NTSR1 immunoreactivity are present in 60% of lung adenocarcinoma tumor specimens [92]. Lung cancer patients with high NTSR1 have decreased relapse-free survival than patients with low NTSR1. High NTSR1 expression results in poor prognosis of patients with ductal breast cancer, prostate cancer, or head and neck squamous carcinoma [93,94]. Gefitinib or SR48692 inhibits the growth of lung cancer cells [30]. Gefitininb synergizes with SR48692 at inhibiting lung cancer proliferation. Similarly, lapatinib inhibits the growth of lung cancer cells and is potentiated by SR48692 [31]. The results indicate that the action of TKIs can be potentiated by GPCR antagonists.

## 4. Transactivation

Transactivation occurs in discrete domains of the plasma membrane, termed lipid rafts. Lipid rafts contain cholesterol, glycolipids, and myelin derivatives and form domains containing GPCRs and RTKs [95]. The ability of NTSR1 to transactivate the EGFR occurs within minutes. After EGFR phosphorylation, it localizes to clathrin-coated pits and is internalized by coated vesicles [96]. The GPCR is in close proximity to the RTK in the lipid rafts [97]. After transport to endosomes, the EGFR can be degraded in lysosomes or recycled to the plasma membrane [98].

Adding NTS to NSCLC cells causes the release of TGFα, leading to increases in phosphorylation of tyrosine^1068^ of the EGFR [30]. NTSR1 regulates EGFR transactivation in several cancer cells [99,100,101]. Adding NTSR1 siRNA to NSCLC cells impairs phosphorylation of tyrosine^1068^-EGFR [30]. Table 1 shows that NTSR1 regulates EGFR transactivation in gastric, head and neck, neuroendocrine, NSCLC, and prostate cancer cells. Transactivation is enhanced by MMP and ROS but inhibited by TKI or SR48692. NTSR1 regulates transactivation of HER2 and HER3 in NSCLC cells, leading to ERK and AKT activation, increasing cancer growth and survival.

Adding NTS to NSCLC cells increases ROS, which is inhibited by SR48692 or diphenylene iodonium (DPI), an inhibitor of nicotinamide adenine dinucleotide phosphate oxidase (NOX) and dual oxidase (DUOX) enzymes (Table 2). NOX enzymes catalyze the following reaction:

NADPH + 2O_2_ → NADP^+^ + 2O^2−^ + H^+^

**Figure 4:**
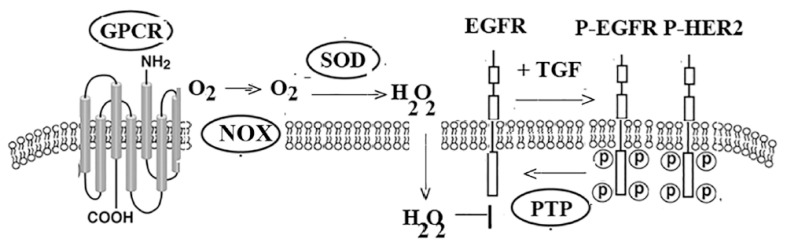
ROS species. Adding GPCR ligand causes activation of NOX which metabolizes oxygen (O_2_) to superoxide (O_2_^−^.). Extracellular superoxide is metabolized by superoxide dismutase to hydrogen peroxide (H_2_O_2_) which diffuses into the cytosol and inhibits protein tyrosine phosphatase (PTP). TGFα increases the tyrosine phosphorylation of the EGFR. The EGFR forms heterodimers with HER2.

Figure 4 shows that activated GPCRs increase NOX activity, which metabolizes oxygen to superoxide. Extracellular superoxide is metabolized to hydrogen peroxide by superoxide dismutase. Hydrogen peroxide enters the cytosol where it oxidizes cysteine amino acids in protein tyrosine phosphatase, inhibiting its activity. Protein tyrosine phosphatase removes the phosphate from tyrosine on RTKs; however, its activity is impaired by ROS. Adding NTS to NSCLC cells significantly increases phosphotyrosine^1068^-EGFR and phosphotyrosine^1248^-HER2 and ROS by 355, 285, and 228%, respectively. Table 2 shows that the increase in ROS, EGFR, and HER2 phosphorylation is impaired by SR48692 or DPI.

It remains to be determined if NTS regulates the oxidation of cysteine amino acids in Src homology-2 tyrosine phosphatase, resulting in reduced enzymatic activity [102]. Alternatively, NTS could oxidize cysteine^797^ of the EGFR, increasing protein kinase activity [103]. Because EGFR/HER2 heterodimers form, NTSR1 regulates the transactivation of HER2 in an ROS-dependent manner [31]. Adding DPI to NSCLC cells impairs the ability of NTSR1 to regulate RTK transactivation.

While NTSR1 regulates EGFR, HER2, and HER3 transactivation, it is unknown if it affects HER4. Adding bombesin (BB) to NSCLC cells increases phosphotyrosine^1284^HER4 significantly [104]. BB binds to BB_2_Rs and is antagonized by PD176252 or BW2258U89. Adding PD176252 or ibrutinib (TKI) impaired the ability of BB to increase phosphotyrosine^1284^-HER4. Adding BB to NSCLC cells increases tyrosine phosphorylation of the EGFR, HER2, and HER3 [105,106]. Additional research is needed on how GPCRs transactivate HER4 in cancer cells.

Many GPCRs cause transactivation of the ErbB RTKs. Adding vasoactive intestinal peptide to breast cancer cells stimulates EGFR and HER2 tyrosine phosphorylation [107]. VIP interacts with VPAC1 in breast cancer cells, activating Gs. Adding pituitary adenylate cyclase-activating polypeptide (PACAP) to NSCLC cells stimulates EGFR, HER2, and HER3 tyrosine phosphorylation [108,109,110]. PACAP interacts with PAC1 in lung cancer cells, activating Gs and Gq. GPCRs cause transactivation of RTKs, other than those of the ErbB family. Adding WKYMVm to cells causes tyrosine phosphorylation of the HGF receptor [111]. In addition, the formyl peptide receptor 1 regulates transactivation of the TrkA and VEGFR2 [112,113]. GPRCRs regulate the phosphorylation of numerous RTKs.

## 5. Discussion

The transactivation process links GPCRs with RTKs. When a GPCR agonist such as NTS binds to NTSR1, signal transduction occurs through Gαq, causing phosphatidylinositol turnover. SRC and MMP are activated, releasing growth factors. When TGFα binds to the EGFR, homodimers or heterodimers form with other RTKs, such as HER2, HER3, and HER4. Adding NTS to NSCLC cells increases tyrosine phosphorylation of protein substrates, such as phosphatidylinositol-3-kinase and tyrosine amino acids, near the C-terminal of the EGFR. The transactivation process occurs rapidly after adding NTS to cancer cells, and when tyrosine^1068^ of the EGFR is phosphorylated, the MEK/ERK pathway is activated, leading to increased cancer cellular proliferation. Adding NTS to certain NSCLC cells increases phosphotyrosine^1248^HER2 and phosphotyrosine^1289^HER3, leading to the activation of ERK and phosphatidylinositol-3-kinase. The transactivation process is impaired by MMP and SRC inhibitors, such as GM6001 or PP2; however, these agents inhibit signal transduction in normal cells as well as cancer cells.

Because GPCRs and ErbB RTK are overexpressed in cancer relative to normal cells, they can serve as molecular targets for diagnosis and treatment. It will be important to determine which GPCRs and ErbB RTKs are enriched in a patient’s tumor relative to normal cells. If NTSR1 is overexpressed, SR48692 may be used to treat the patient. If the EGFR is mutated, osimertinib may be used to treat the patient. If both NTSR1 and EGFR are enriched in the tumor cells, SR48692 and osimertinib may be used.

The transactivation of RTKs by GPCRs in cancer occurs in an ROS-dependent manner. Oxidative stress is important in carcinogenesis and cancer progression [114]. NOX metabolizes extracellular oxygen to superoxide, which is then metabolized to hydrogen peroxide by superoxide dismutase [115]. ROS can oxidize nucleic acids, lipids, glucose, and proteins, such as the EGFR or protein tyrosine phosphatase [116]. The net result is that NTS increases tyrosine phosphorylation of ErbB RTKs. NOX2 is inhibited by DPI and the related analog ebselen [117]. Nox2ds-tat is a peptide that inhibits the assembly of NOX2 subunits, reducing the ability of GPCR agonists to produce superoxide [118]. The NOX2 inhibitors reduce the growth of cancer cells in vitro; however, it remains to be determined if they will be effective at inhibiting tumor growth in vivo. One goal is to synthesize new NOX2 inhibitors, which are selective for cancer but not normal cells.

## 6. Conclusions

NTSR1 regulates transactivation of the EGFR, HER2, and HER3 in NSCLC cells. The growth of these cells is impaired by TKIs and NTSR1 antagonists. Gefitinib and SR48692 are synergistic at inhibiting NSCLC growth and RTK transactivation in vitro. It remains to be determined if TKIs and GPCR antagonists are synergistic at inhibiting NSCLC growth in vivo.

## Figures and Tables

**Table 1:** Mediated transactivation.

RTK Activated	Mechanism	Cancer Type	Reference
EGFR	MMP	Head and neck cancer	[93]
EGFR	Arachidonic acid release	Prostate cancer	[94]
EGFR	TKI inhibit	Neuroendocrine	[100]
EGFR	β–χατενιν	Gastric	[99]
EGFR	ERK	NSCLC	[30]
HER2	ROS	NSCLC	[31]
HER3	ERK + AKT	NSCLC	[32]
EGFR, HER2, HER3	TKI inhibit	NSCLC	[101]

The mechanism and cancer type is indicated for NTSR1 regulation of ErbB RTK transactivation.

**Table 2:** Effect of ROS.

Addition	% ROS	% PY^1068^-EGFR	%PY^1248^-HER2
None	100 + 7	100 + 8	100 + 6
NTS	228 + 9 **	355 + 26 **	285 + 19 **
NTS + DPI	117 + 15	112 + 6	107 + 7
NTS + SR48692	108 + 6	106 + 7	111 + 5

NCI-H838 cells were treated with 5 μM DPI or 5 μM SR48692 and 0.1 μM NTS added. The % ROS, PY^1248^-HER2 and PY^1068^-EGFR is indicated. The mean value ± S.D. of 4 determinations is shown; *p* > 0.01, ** by ANOVA. NCI-H838 cells have mRNA for NOX1, NOX2, NOX3, NOX5, DUOX1 and DUOX2.

## Data Availability

Not applicable.
